# Co-Expression of IL-7 Improves NKG2D-Based CAR T Cell Therapy on Prostate Cancer by Enhancing the Expansion and Inhibiting the Apoptosis and Exhaustion

**DOI:** 10.3390/cancers12071969

**Published:** 2020-07-20

**Authors:** Cong He, Ying Zhou, Zhenlong Li, Muhammad Asad Farooq, Iqra Ajmal, Hongmei Zhang, Li Zhang, Lei Tao, Jie Yao, Bing Du, Mingyao Liu, Wenzheng Jiang

**Affiliations:** Shanghai Key Laboratory of Regulatory Biology, School of Life Sciences, East China Normal University, Shanghai 200241, China; hecong0126@126.com (C.H.); zoeyzhouying@126.com (Y.Z.); lzlecnu@163.com (Z.L.); asadfarooq601@yahoo.com (M.A.F.); iqraasad1263@yahoo.com (I.A.); zhanghongmei0501@126.com (H.Z.); 15221152917@163.com (L.Z.); taol1988@126.com (L.T.); ecnuyaojie@163.com (J.Y.); bdu@bio.ecnu.edu.cn (B.D.)

**Keywords:** NKG2D, CAR T, IL-7, prostate cancer, cell therapy

## Abstract

Chimeric antigen receptor (CAR) T-cell therapy is a promising approach in treating solid tumors but the therapeutic effect is limited. Prostate cancer is a typical solid malignancy with invasive property and a highly immunosuppressive microenvironment. Ligands for the NKG2D receptor are primarily expressed on many cancer cells, including prostate cancer. In this study, we utilized NKG2D-based CAR to treat prostate cancer, and improved the therapeutic effect by co-expression of IL-7. The results showed that NKG2D-CAR T cells performed significantly increased cytotoxicity against prostate cancer compared to non-transduced T cells in vitro and in vivo. Moreover, the introduction of the *IL-7* gene into the NKG2D-CAR backbone enhanced the production of IL-7 in an antigen-dependent manner. NKG2DIL7-CAR T cells exhibited better antitumor efficacy at 16 h and 72 h in vitro, and inhibited tumor growth in xenograft models more effectively. In mechanism, enhanced proliferation and Bcl-2 expression in CD8^+^ T cells, decreased apoptosis and exhaustion, and increased less-differentiated cell phenotype may be the reasons for the improved persistence and survival of NKG2DIL7-CAR T cells. In conclusion, these findings demonstrated that NKG2D is a promising option for CAR T-cell therapy on prostate cancer, and IL-7 has enhanced effect on NKG2D-based CAR T-cell immunotherapy, providing a novel adoptive cell therapy for prostate cancer either alone or in combination with IL-7.

## 1. Introduction

The application of genetic redirection of T lymphocytes with chimeric antigen receptors (CARs) in cancers has re-energized the field of cancer immunotherapy. The tremendous success of CAR T cells in eradicating CD19-expressing acute and chronic B cell leukemias has attracted more attention in applying CARs to solid tumors [1,2]. However, several limitations need to be resolved to extend the success to solid tumors [3]. The key limitations are the accumulation and survival of transferred CAR T cells in the immunosuppressive tumor microenvironment, which can impair the proliferative ability and in vivo persistence of the infused T cells [4].

Prostate cancer is the second most frequent cancer among males, leading to a huge burden of incidence and mortality in the world [5]. Once symptoms appear, it is mostly diagnosed as a progressive prostate cancer, and approximately one in five patients of metastatic castration-resistant prostate cancer (mCRPC) dies annually [6]. Given that metastatic prostate cancer is associated with an unfavorable prognosis and poses enormous therapeutic challenges, any novel strategy of effective treatment developed for this advanced disease is a top priority for the scientists nowadays [7].

Ligands for the natural killer group 2 member D (NKG2D), an NK-cell activating receptor [8], are primarily expressed on most types of tumor cells, including hematological and solid tumors, but are normally absent or expressed in low levels on healthy tissues [9,10]. Several kinds of NKG2D-based CARs have been developed and their extensive therapeutic effects on various tumors have been studied [11]. Here, we identified NKG2DLs-expressing prostate cancer as being susceptible to NKG2D-CAR T cell-mediated attack, providing a new strategy for effective treatment.

NKG2D-CAR T cells have shown safety in clinical trials for treating patients with multiple hematological and solid tumors, and although complete alleviation has been achieved in selected patients, higher efficacy still remains desirable. The limited expansion of CAR T cells in vivo is one of the obstacles that need to be overcome to boost clinical efficacy [12]. Transgenic expression of growth-promoting cytokines (e.g., IL-15, IL-12) in CAR T cells represents a strategy to support the long-term expansion and persistence [13,14]. IL-7 has been applied to augment the T-cell antitumor immune response as a T-cell growth factor [15,16]. Therefore, we further modified NKG2D-CAR T cells to secrete IL-7, a stimulatory cytokine known to improve the proliferation and survival of T cells [17,18]. Our study demonstrated that NKG2D-CAR T-cell treatment effectively inhibited the growth of prostate cancer. Furthermore, transgenic expression of IL-7 enhanced the proliferative, persistence and anti-prostate cancer activity of NKG2D-CAR T cells in vitro and in vivo.

## 2. Results

### 2.1. NKG2D-CAR T Cells Effectively Recognize and Lyse NKG2DLs^+^ Prostate Cancer Cell Lines In Vitro and Co-Expression of IL-7 Enhances Its Activation and Function

**Figure 1:**
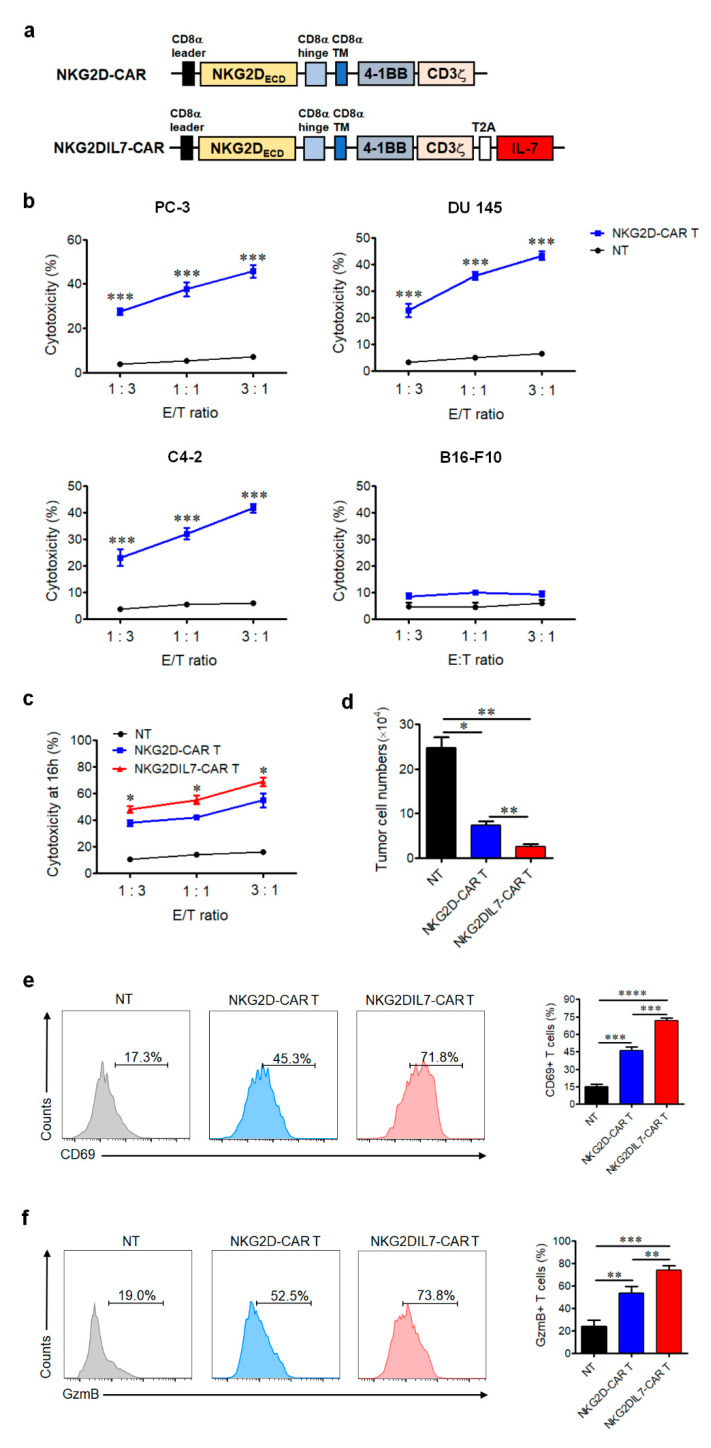
NKG2DIL7-chimeric antigen receptor (CAR T cells display enhanced antitumor activity in vitro. (**a**) Domain architecture of engineered NKG2D-CAR and NKG2DIL7-CAR constructs. (**b**) Cytotoxic activity of NKG2D-CAR or non-transduced T cells against prostate cancer cell lines was determined by Annexin-V staining. B16-F10 melanoma cells served as negative target cell control. The effector cells were co-cultured for 4 h with target cells at E:T ratio of 1:3, 1:1 and 3:1, respectively. (**c**,**d**) Cytotoxic assays were determined by Annexin-V staining at 16 h of co-culture of NKG2D-CAR or NKG2DIL7-CAR T with PC-3 at E:T ratios of 3:1,1:1 and 1:3. E (**c**), PC-3 tumor cell viability assay was performed after 72 h of co-culture with non-transducted T (NT), NKG2D-CAR or NKG2DIL7-CAR T cells at E:T ratio of 3:1 (**d**). (**e**,**f**) Flow cytometric analysis of CD69 and granzyme B in T cells after the stimulation of tumor cells. * *p* < 0.05, ** *p* < 0.01, *** *p* < 0.001, **** *p* < 0.0001. Data are representative of greater than three independent experiments.

We synthesized NKG2D-CAR construct consisting of the extracellular part of NKG2D, linked to the intracellular signaling domains of 4-1BB and CD3ζ molecule via a CD8α hinge-transmembrane domain (Upper panel of Figure 1a). CD3/CD28-activated T cells separated from healthy donors were transduced with lentivirus expressing NKG2D-CAR, and the transduction efficiency was identified by staining of the anti-human NKG2D antibody. To determine whether NKG2D-CAR T cells can recognize and lyse prostate cancer cells such as PC-3, DU 145 and C4-2, which expresses a high level of NKG2DLs (Additional file 1: Figure S1), the cytotoxicity of NKG2D-CAR T cells against the NKG2DLs^+^ prostate cancer cells was determined by detection of the apoptosis of the target cells. The results showed that NKG2D-CAR T cells exhibited significant cytolytic activity against several prostate cancer cell lines in an E:T ratio-dependent manner, but had no killing effect on the NKG2DLs^−^ cell line B16-F10 (Figure 1b). Therefore, NKG2D-based CAR T cells could specifically and efficiently kill NKG2DLs^+^ prostate cancer cells in vitro.

To generate NKG2D-CAR T cells expressing IL-7 (NKG2DIL7-CAR T cells), we modified T cells with a lentivirus vector encoding *IL-7* on NKG2D-CAR backbone as shown in the schematic diagram (Lower panel of Figure 1a). IL-7 linked to NKG2D-CAR with 2A peptide could be secreted outside the cells. T cells separated from PBMCs were transduced with lentivirus NKG2DIL7-CAR. Transduction efficiency was determined by FACS analysis (Additional file 2: Figure S2). To determine the killing ability of NKG2DIL7-CAR T cells against prostate cancer cells, prostate cancer cell line PC-3 was used as target cells and the cytotoxicity assay was performed at different E:T. The results showed that both of two CAR T cells had a significant cytotoxic effect on PC-3 cells and NKG2DIL7-CAR T cells exhibited better antitumor efficacy than conventional NKG2D-CAR T cells at 16 h (Figure 1c) and 72 h (Figure 1d), demonstrating that the killing capacity of NKG2D-based CAR T cells could be enhanced by co-expressing of IL-7.

We next explored the expression of CD69, a sensitive activation marker for T-cell function [19,20]. A higher level of CD69-positive cells was observed in both types of CAR T cells compared to non-transduced T cells in response to PC-3 tumor cells. However, a higher level of CD69 expression was detected in NKG2DIL7-CAR T cells (Figure 1e).

Furthermore, granzyme B is also pivotal for cytolytic function of CAR T cells [21,22]. The results demonstrated that NKG2D-CAR T cells produced more granzyme B than non-transduced T cells when co-cultured with target cells and transgenic expression of IL-7 into conventional NKG2D-CAR T cells could significantly enhance the expression of granzyme B (Figure 1f).

### 2.2. Co-Expression of IL-7 Enhances the Proliferation of NKG2D-CAR T Cells

**Figure 2:**
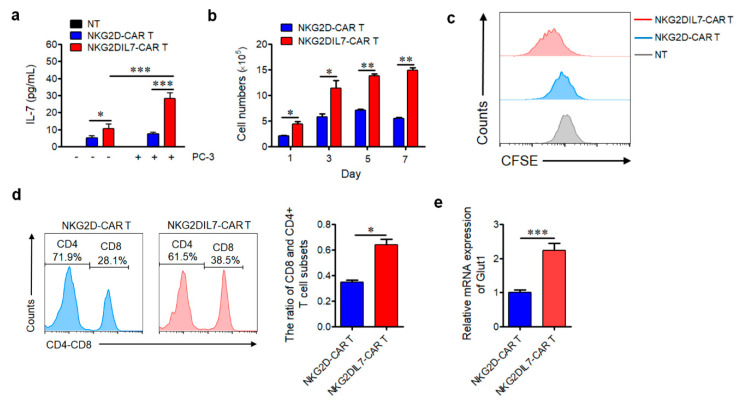
Co-expression of IL-7 enhances the proliferation of NKG2D-CAR T cells. (**a**) NKG2D-CAR or NKG2DIL7-CAR T cells were cultured in the absence or presence of PC-3 tumor cells at E:T ratio of 3:1 for 24 h without any exogenous cytokines, and the co-culture supernatants were detected for concentrations of IL-7 by ELISA. (**b**) Expansion of NKG2D-CAR and NKG2DIL7-CAR T cells after stimulation with tumor cells. The number of initial CAR T cells was 2.5 × 10^5^, and cell numbers were measured by Vi-CELL every other day. (**c**) Non-transduced, NKG2D-CAR and NKG2DIL7-CAR T cells were labeled with 5(6)-carboxyfluorescein diacetate succinimidyl ester (CFSE) before being stimulated by PC-3 tumor cells, the dilution of CFSE was determined by flow cytometry after 7 days of co-culture. (**d**) The flow cytometric analysis of the percentage and ratio of CD8^+^ and CD4^+^ T cells in vitro on 7th day after stimulation, the initial CD4 and CD8 percentages were the same. (**e**) Effector and target cells were co-cultured at E:T ratio of 2:1 for 24 h, and the expression of Glut1 was measured by quantitative real-time PCR. * *p* < 0.05, ** *p* < 0.01, *** *p* < 0.001. Data are representative of 4 donors and have been gated on NKG2D^+^ T cells.

To validate the expression of IL-7, NKG2DIL7-CAR, NKG2D-CAR and non-transduced T cells were cultured in media with or without tumor cells for 24 h. The supernatants were collected to determine the secretion of IL-7. We found that NKG2DIL7-CAR T cells produced a relatively greater amount of IL-7 compared with conventional CAR T cells in the absence of a tumor (Figure 2a). Surprisingly, a robust increase of IL-7 expression was observed in NKG2DIL7-CAR T cells when co-cultured with PC-3 cells. These results indicated that the production of IL-7 was dependent on the presence of target cells.

To define the effect of IL-7 on the proliferation of NKG2D-CAR T cells, non-transduced T cells and two types of transduced CAR T cells were stimulated with target cells (PC-3). Cell numbers were measured by Vi-CELL every other day and CFSE-based assay was performed to assess the proliferation ability. The results showed that co-expression of IL-7 induced significantly greater expansion of the numbers of CAR T cells when compared with its counterpart on day 1, day 3 until day 7 (Figure 2b). Moreover, NKG2DIL7-CAR T cells displayed greater proliferation potential after 7 days of culture compared with NKG2D-CAR and non-transduced T cells (Figure 2c). The data of T cell subsets of CD4^+^ and CD8^+^ T cells revealed a pronounced increase in CD8^+^ T cell numbers, resulting in an obvious increase in CD8/CD4 ratio in NKG2DIL7-CAR transduced T cells compared with NKG2D-CAR T cells (Figure 2d).

IL-7 can promote glucose transporter (Glut1) trafficking and glucose uptake to support cell survival [23]. To determine whether overexpression of IL-7 likewise affected Glut1 expression in NKG2D-CAR T cells, CAR T cells were stimulated with antigen cells for 24 h and mRNA level of Glut1 was detected (Figure 2e). Compared with NKG2D-CAR T cells, a higher mRNA level of Glut1 was detected in NKG2DIL7-CAR T cells, demonstrating that higher Glut1 transport may be an important factor for IL-7 to improve the survival of NKG2D-CAR T cells.

### 2.3. Transgenic Expression of IL-7 Reduces the Apoptosis of NKG2D-CAR T Cells

**Figure 3:**
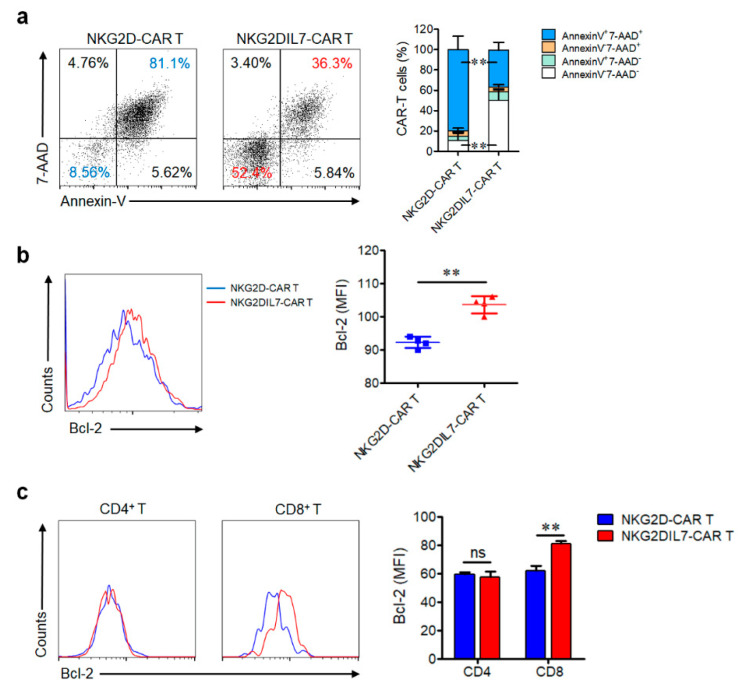
Co-expression of IL-7 reduces the apoptosis of NKG2D-CAR T cells. (**a**) After NKG2D-CAR and NKG2DIL7-CAR T cells were cocultured with PC-3 tumor cells for 7 days, the cells were stained with Annexin-V/7-AAD and the apoptosis was detected by FACS. The statistical analysis was shown in the right panel. (**b**) Flow cytometric analysis of Bcl-2 protein expression in CAR T cells 7 days after tumor cell stimulation. (**c**) The Bcl-2 expression in CD4^+^ and CD8^+^ CAR T cells was detected by FACS 7 days after tumor cell stimulation. ** *p* < 0.01. Data are representative of 4 donors and have been gated on NKG2D^+^ T cells.

The role of IL-7 in survival and anti-apoptosis was verified in several studies previously [17,24]. To figure out whether co-expression of IL-7 enhanced the anti-apoptosis ability of CAR T cells, NKG2D-CAR and NKG2DIL7-CAR T cells were cocultured with PC-3 tumor cells without IL-2 for 7 days and the apoptosis cells was detected by Annexin-V/7AAD staining. The results showed that the cells apoptotic rate in NKG2DIL7-CAR T cells (36 ± 8%) was significantly lower than NKG2D-CAR T cells (81.1 ± 15%) (Figure 3a).

Bcl-2 is a downstream protein of IL-7-mediated signal pathways [24,25]. The up-regulation of Bcl-2 is related to improved anti-apoptosis and survival ability [26]. FACS analysis of Bcl-2 expression illustrated that NKG2DIL7-CAR T cells expressed significantly higher amounts of anti-apoptotic protein Bcl-2 compared with NKG2D-CAR T cells (Figure 3b). In addition, further analysis of Bcl-2 expression in T cell subsets showed that there was no difference in CD4^+^ T cells, but a significantly higher expression was observed in CD8^+^ NKG2DIL7-CAR T cells (Figure 3c). 

### 2.4. IL-7 Preserves Less Differentiated Cell Phenotype and Inhibits the Exhaustion of CAR T Cells

**Figure 4:**
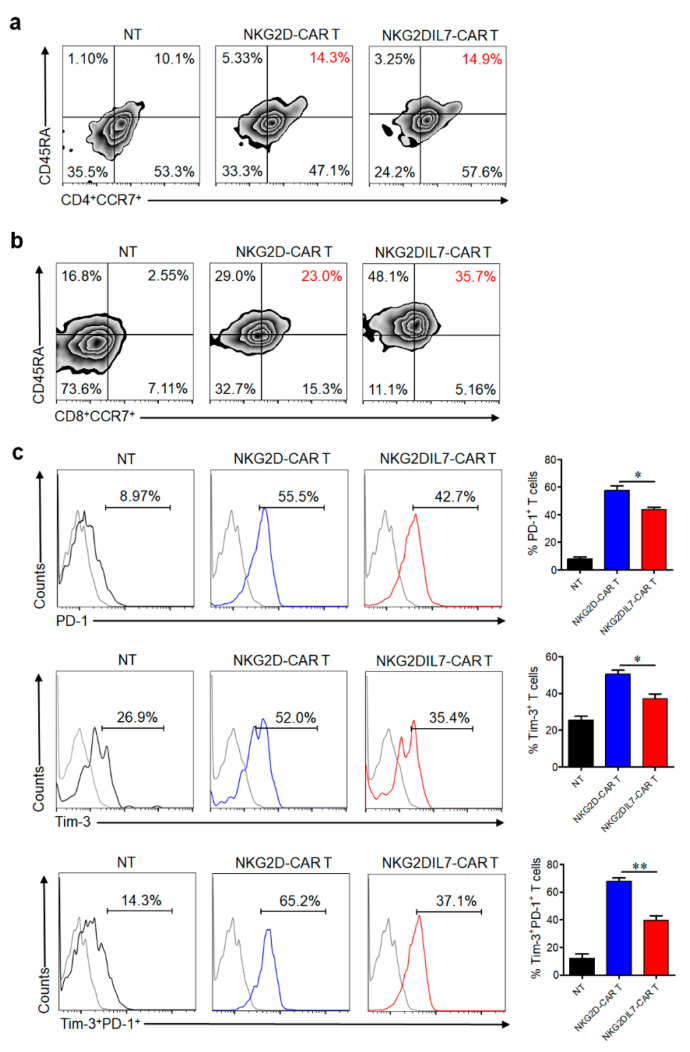
IL-7 increases CAR T cells with the less differentiated phenotype and inhibits CAR T-cell exhaustion. (**a**,**b**) T-cell phenotype based on CD45RA and CCR7 expression in CD4^+^ (**a**) and CD8^+^ (**b**) CAR T cells was analyzed by FACS after 14 days of culture. (**c**), Surface expression of PD-1 and Tim-3 on CAR T cells was analyzed by FACS after 7 days of co-culture with tumor cells. * *p* < 0.05, ** *p* < 0.01. Data are representative of four T-cell lines.

T cells usually exist several phenotypes, which have distinct proliferation, survival, and effector capabilities [27,28,29]. To determine whether overexpression of IL-7 influenced the differentiation of CAR T cells, NKG2D-CAR and NKG2DIL7-CAR T cells were cultured for 14 days, and were stained with CD45RA and CCR7 to analyze T cell subsets. A comparable proportion of CCR7^+^CD45RA^+^ subset in CD4^+^ T cells, but a remarkable increased CCR7 and CD45RA double-positive cells in CD8^+^ T cells were observed after co-expression of IL-7 (Figure 4a,b). T cells that express CD45RA and CCR7 are a group of less differentiated cells, which correlate with CAR-T cell expansion, survival and long-term persistence [30]. Our results suggested that IL-7 could preserve a less differentiated phenotype of CD8^+^ T cells, which might be beneficial for the future clinical application of CAR T cell therapy.

Cancer cells can inhibit the functions of T cells by expressing the ligands of inhibitory receptors such as PD-1 and Tim-3 [31,32], leading to T cell exhaustion. After 7 days of co-cultured with tumor cells, NKG2DIL7-CAR T cells exhibited lower expression of PD-1 and Tim-3, especially in Tim3^+^ PD1^+^ expression (Figure 4c), suggesting that IL-7 could prevent CAR-T cell exhaustion and protect T cells from deleterious immunosuppressive actions of tumor cells. 

### 2.5. NKG2D-CAR T Cells Expressing IL-7 Have Improved Antitumor Activity against Xenograft Prostate Tumor Model

**Figure 5:**
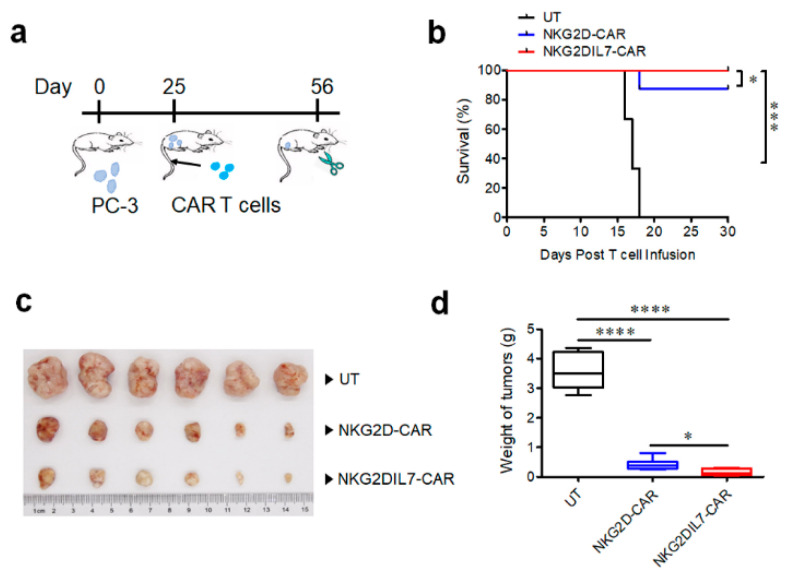
Engineering expression of IL-7 enhances the antitumor activity of NKG2D-CAR T cells in the xenograft prostate tumor model. (**a**) A schematic of in vivo experiment. (**b**) Kaplan-Meier survival analysis of PC-3 challenged mice after treatment with CAR-T cells. (**c**,**d**) The photograph of residual tumors (**c**) and the tumor weight (**d**) of the mice treated with NKG2D-CAR and NKG2DIL7-CAR T cells at the endpoint of the experiments. * *p* < 0.05, *** *p* < 0.001, **** *p* < 0.0001, *n* = 6.

Finally, we evaluated the ability of two types of CAR T cells against PC-3 prostate tumor cells in a xenograft mouse model. NSG mice were engrafted s.c. with 2 × 10^6^ tumor cells. Once the tumor volumes had reached approximately 150–200 mm^3^ size, non-transduced T cells, NKG2D-CAR and NKG2DIL7-CAR T cells (1 × 10^7^ cells/mouse) were injected i.v. into the tumor-bearing mice (Figure 5a). All the mice of NT group had to be euthanized due to large tumor volume on day 18, NKG2D-CAR T cells produced remarkable antitumor ability in vivo compared with NT group, and the survival rate of tumor-bearing mice was more than 80% (Figure 5b). Furthermore, the tumor volume and weight of the group treated with NKG2D-CAR T cells reduced significantly compared to the control group. Interestingly, the tumor volume and weight were lower in the group treated with NKG2DIL7-CAR T cells than that with NKG2D-CAR T cells (Figure 5c,d), demonstrating that NKG2D-CAR T-cell therapy on prostate cancer could be an efficient method and introduction of IL-7 into NKG2D-CAR T cells can enhance antitumor ability in vivo.

**Figure 6:**
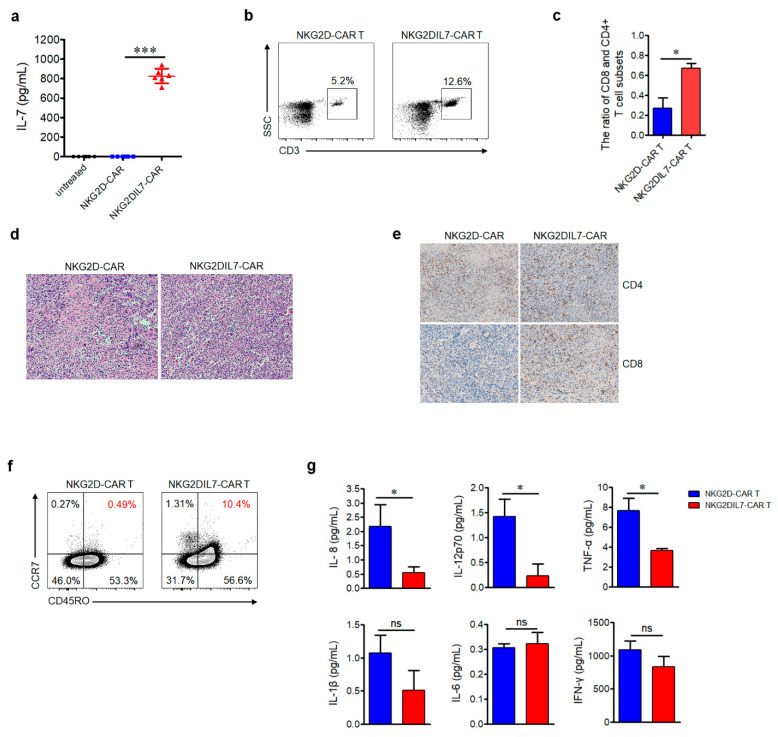
Engineering expression of IL-7 increases T cell accumulation and central memory-T cell subsets in the tumor site. (**a**) The level of IL-7 in sera of mice was detected by ELISA. (**b**,**c**) Detection of human T cells and the subsets of CD4^+^ and CD8^+^ T cells in the blood 25 days after CAR T cells adoptive transfer. (**d**,**e**) Tumor tissues were removed from the mice 25 days after treatment with CAR-T cells, and each tissue was divided into two parts. One part was stained with H&E (**d**), the other part was used for immunohistochemistry (IHC) (**e**). For IHC, combinations of anti-CD4 antibody and anti-CD8 antibody. The images were obtained under ×100 magnification. (**f**) Detection of Tcm phenotype of CAR T cells in the tumor site at the endpoint of the experiments. (**g**) The levels of IL-1β, IL-6, IL-8, IL12p70, IFN-γ and TNF-α in mouse serum were evaluated by a CBA kit. ns, not significant, * *p* < 0.05, *** *p* < 0.001. Data shown are representative of 6 mice per group from 2 independent experiments.

The sera of mice were collected to determine the IL-7 cytokine level, the results showed that a high level of IL-7 was detected in the NKG2DIL7-CAR treatment group but not in the other two groups (Figure 6a). T cells in the blood of mice were analyzed by flow cytometry, the data indicated that the proportion of T cells in the group treated with NKG2DIL7-CAR T cells was significantly higher than that with NKG2D-CAR T cells (Figure 6b). The proportion of CD4^+^ and CD8^+^ T cells in blood was also analyzed by FACS and more CD8^+^ T cells have been detected in the mice treated with NKG2DIL7-CAR T cells (Figure 6c). 

To analyze the persistence and accumulation of CAR T cells at the tumor site, the tumors were excised after the treatment and the histopathological analysis was performed. H&E staining results showed that there were more T cells in the tumor sections of the mice treated with NKG2DIL7-CAR T cells compared with the conventional CAR-T group (Figure 6d). Furthermore, immunohistochemistry staining analysis indicated that there were more CD8^+^ T cells in the tumor sections of the NKG2DIL7-CAR treatment group (Figure 6e), all of which were consistent with the results in vitro. 

To address whether IL-7 affected the T cell phenotype in vivo, CD45RO and CCR7 on T cells, the markers of central memory T cells (Tcm), were examined by flow cytometry. The results revealed a higher proportion of Tcm-phenotypic cells in the tumor tissue from the mice treated with NKG2DIL7-CAR T cells (Figure 6f), suggesting that IL-7 might be beneficial to the formation of central memory T cells. Moreover, there were fewer cytokines such as IL-8, IL-12, TNF-α in the blood from the mice treated with NKG2DIL7-CAR T cells (Figure 6g). Collectively, our in vivo results elucidated that NKG2D-based CAR T cells could effectively kill prostate cancer cells, and co-expression of IL-7 could enhance the antitumor function of NKG2D-CAR T cells against prostate cancer.

## 3. Discussion

For treating B-cell malignancies, the second and third-generation CAR T cells have gained much success [14,33], but its efficacy to treat solid tumors still remains insufficient [3,4,34], particularly due to poor proliferation and survival of CAR T cells in vivo. NKG2D-based CAR T cells for immunotherapies have been reported to be promising for targeting NKG2D ligand-positive cancers [11,35]. In addition, NKG2D ligands are also expressed in tumor blood vessels, myeloid cells, immunosuppressive cells (such as Tregs and MDSCs) and endothelial cells in the tumor microenvironment [36], indicating that NKG2D-CAR could not only target tumors but also other cells in the microenvironment that promote tumor progression. To the best of our knowledge, there were few reports of targeting prostate cancer with NKG2D-CAR T cells. Here, we found that NKG2D ligands were highly expressed on human prostate cancer cell lines, and our designed NKG2D-based CAR T cells could effectively recognize and lyse NKG2D ligand-positive prostate cancer cells. 

CAR T-cell immunotherapy for prostate cancer is extremely promising [37,38], but a major challenge that needs to be addressed is enhancing the proliferation and survival of CAR T cells in the highly immunosuppressive microenvironment. Previous studies have characterized that incorporation of CD137 (4-1BB) signaling domain into CARs rather than CD28 domain can improve the persistence and antitumor ability in vivo [39,40,41]. Nevertheless, in the highly immunosuppressive microenvironment of metastatic prostate cancer, the second generation of CAR with a 4-1BB signaling domain may not provide sufficient co-stimulation for T cells. Therefore, co-expression of a cytokine such as IL-7 in CAR T cells may be a valid way to improve the persistence and antitumor activity of CAR T cells in vitro and in vivo.

In the present study, there was no major difference in IL-7 production between NKG2D-CAR and NKG2DIL7-CAR T cells in the absence of tumor, but when co-cultured with PC-3 cells, IL-7 expression in NKG2DIL7-CAR-transduced T cells increased vigorously. Thus, our current approach provided cytokine IL-7 directly to the tumor site, which could possibly avoid toxicities reported with high dose systemic cytokine administration [42,43]. Previously, it has been reported that the constitutive expression of IL-7 has led to increased T-cell accumulation in vitro by enhancing T cell proliferation and survival. The author demonstrated that overall enhanced tumor rejection was due to improved T cell expansion rather than upregulation of effector function [44]. In another study, it has been reported that transgenic expression of IL-7 could also enhance the effector function of CAR T cells both in vitro and in vivo [45]. Here we validated that transgenic expression of IL-7 could increase the effector function as well as promote the expansion at the tumor site. The expression of T cell exhaustion markers such as PD-1 and TIM3 were also downregulated by IL-7 transgenic expression, which also pointed towards enhanced T cell effector function. 

NKG2DIL7-CAR T cells exhibited greater cell numbers and cell viability than conventional NKG2D-CAR T cells on day 7. Our analysis indicated that co-expression of IL-7 could enhance both proliferation and survival of NKG2D-CAR T cells. Elevated expression of Bcl-2, an anti-apoptotic protein [46], and Glut1 in NKG2DIL7-CAR T cells further supported our claim. In vitro expression analysis of Bcl-2 in CD4 and CD8 populations further revealed that expansion in T cell numbers was mainly due to a rise in CD8^+^ T cells, but not CD4^+^ T cells. Less differentiated CD8^+^ T cell subsets i.e., naïve and T memory stem cells have been recognized recently to be critical for expansion, survival and long-term persistence in vivo [30,47]. Interestingly, in the present study CD8^+^ T cells displayed CD45RA^+^ CCR7^+^ phenotype in vitro, which was also beneficial for in vivo antitumor efficacy.

Consistent with in vitro results, there were more CAR T cells, especially CD8^+^ T cells, in peripheral blood from the mice treated with NKG2DIL7-CAR T cells, which might be due to a more plentiful CD8^+^ population after in vitro culture. Moreover, the histological analysis indicated that CAR T cells could infiltrate into the tumor tissues and there was more T cell infiltration, especially CD8^+^ T cells, in tumor tissues treated with NKG2DIL7-CAR T cells. Increased CD8^+^ T cells will be more effective for adoptive T cell immunotherapy in prostate cancer patients [48].

We found increased expression of CD45RO and CCR7 with transgenic expression of IL-7 in vivo, representing central memory T cell (Tcm) phenotype. As we know, Tcm is beneficial for adoptive T cell transfer because it provides instant antitumor immunity to patients and endows them with immune memory to prevent cancer recurrence [28]. Interestingly, there were fewer cytokines such as IL-8, IL-12, TNF-α in the blood of the mice treated with NKG2DIL7-CAR T cells, indicating that NKG2DIL7-CAR T therapy was relatively safe. 

The limitation in our study is that human NKG2D-based CAR we used does not recognize murine NKG2D ligands, so the associated potential toxicity in the current mouse model could not be assessed. Besides, the effects of IL-7 for NKG2D CAR T cell functions were evaluated in the absence of Tregs, MDSCs, M2-macrophages and immune checkpoint molecules which created an immune-suppressive environment in cancer tissues. In our study, there is no evidence that IL-7 plays a role in immuno-suppressive microenvironment in prostate cancer tissues of the patients. However, several reports have shown that IL-7 is able to antagonize the immunosuppressive network to improve immune function on cancer cells [49]. For example, Treg cells, which have low expression of CD127 and high level of CD132 on their surface, accumulate in the tumor microenvironment and inhibit immune responses. IL-7 can directly abrogate the Treg-mediated suppression of effector T cell proliferation [50] and decrease the population of Tregs in the lung cancer model [51]. Therefore, we suppose that NKG2D-CAR T cells expressing IL-7 would have the capacity to persist in the immuno-suppressive microenvironment in prostate cancer tissues and induce potent antitumor immunity in patients, but more evidence is needed.

## 4. Materials and Methods

### 4.1. Cell Lines and Culture

The immortalized human embryonic kidney (HEK) -293T cell line was purchased from the American Type Culture Collection (ATCC, Manassas, VA, USA) and used for lentivirus packaging. Prostate cancer cell lines (PC-3, DU 145 and C4-2) were kindly provided by Dr. Zhengfang Yi (East China Normal University, Shanghai, China). The mouse melanoma cell line, B16-F10, was used as an antigen-negative control. The cell lines were cultured and maintained in DMEM (GIBCO, Waltham, MA, USA) supplemented with 10% heat-inactivated FBS, 100 IU/mL penicillin, and 100 mg/mL streptomycin at 37 °C in a humidified atmosphere containing 5% CO_2_.

### 4.2. CAR Construction and Lentivirus Production

We constructed two different human codon-optimized second-generation CARs, which have a specificity against NKG2D ligands on tumors. NKG2D-CAR lentiviral vector consisted of the extracellular portion of human NKG2D (aa 82-216), linked to a CD8α hinge-transmembrane domain along with CD3ζ and 4-1BB signaling domains as described previously [52,53]. Human IL-7 gene (GenBank NM_000880.4) was fused with the NKG2D-CAR by foot-and-mouth disease virus 2A ribosomal skipping sequence to generate NKG2DIL7-CAR. 

High titer replication-defective lentivirus particles were generated by using HEK-293T lentivirus packaging cell line along with packaging plasmid vectors (psPAX2, pMD2.G). On the day before transfection, HEK-293T cells were seeded in a 10 cm culture dish and when the cell fusion rate reached to 80–90%, CAR-encoding vector, psPAX2 and pMD2.G were transfected into HEK-293T cells at a ratio of 5:5:3 with the help of polyethyleneimine (MW 25000) (Polysciences, Warrington, PA, USA) transfection system [54]. Supernatant harvested at 48 h and 72 h post-transfection were concentrated by ultracentrifugation (Beckman Coulter, Brea, CA, USA) for 2.5 h at 25,000 rpm at 4 °C. Viruses were aliquoted and stored at −80 °C until used for experiments. All the experiments performed in this study were from the concentrated virus stock. 

### 4.3. T-Cell Isolation, Modification and Culture

Peripheral blood mononuclear cells (PBMCs) were isolated from healthy volunteer donor cord blood after informed consent under the protocol approved by East China Normal University Internal Review Board. Primary human CD4^+^ and CD8^+^ T cells were positively selected from PBMCs with the CD4 and CD8 MicroBeads (Miltenyi, Bergisch Gladbach, Germany) following the manufacturer’s instructions. Then, CD4^+^ and CD8^+^ T cells were mixed and activated for 48 h using the T Cell TransAct™ (Miltenyi) with the recommended titer of 1:100. Activated T cells were transduced with lentiviruses expressing NKG2D-CAR or NKG2DIL7-CAR supplemented with polybrene (10 µg/mL) and centrifuged for 1 h at 1800 rpm, 28 °C, and then incubated overnight. After 12 h of post-transduction, T cells were washed and cultured in X-VIVO^TM^ 15 medium (Lonza, Switzerland) in the presence of human recombinant IL-2 (rhIL-2, 50 U/mL) and incubated for 2 days. Transduction efficiency was monitored by flow cytometry with APC anti-human NKG2D antibody staining. No exogenous cytokines were added in subsequent experiments.

### 4.4. Flow Cytometry and Antibodies

The following cell surface fluorochrome-conjugated monoclonal antibodies were used to detect T cells phenotype: FITC anti-human CD4, PE/Cy7 anti-human CD8, PE anti-human CD3 (Biolegend, San Diego, CA, USA) and APC anti-human NKG2D (eBioscience). T cell activity was determined by staining the surface APC anti-human CD69, intracellular PE anti-human IFN-γ (BD Biosciences, San Diego, CA, USA), PE anti-human GzmB (eBioscience, San Diego, CA, USA) and PE anti-human Bcl-2 (Biolegend) antibodies. PE anti-human PD-1 and APC anti-human Tim-3 were purchased from Biolegend. FITC anti-human CD45RA, Percp-cy5.5 anti-human CCR7 and PE anti-human CD45RO were purchased from Biolegend to determine T cell subsets. APC Annexin-V and 7-Aminoactinomycin D (7-ADD) from BD Biosciences were used for apoptosis staining. APC mouse IgG1, κ isotype, PE mouse IgG1 isotype, PE/Cy7 mouse IgG2a, κ isotype, FITC mouse IgG1 isotype, PE/Cy7 mouse IgG1, κ isotype (Biolegend) were used as controls. Flow cytometry was performed on the BD LSRFortessa flow cytometer, and data were analyzed using FlowJo Version 10 software.

### 4.5. Cytotoxicity Assays

To evaluate the cytotoxicity of CAR T cells, prostate cancer cell lines were used as target cells when the viability was > 95% on the Vi-CELL counting machine (Beckman Coulter). The target cells were labeled with CFSE (eBioscience), and co-cultured with NKG2D-CAR T cells or NKG2DIL7-CAR T cells at an effector: target ratio of 3:1, 1:1 and 1:3. No exogenous cytokines were added. Cytotoxicity was measured as the percentage of apoptotic target cells. 

Additional cytotoxicity of NKG2D-CAR and NKG2DIL7-CAR T cells was measured by the Vi-CELL counting machine (Beckman Coulter). After the target cells were co-cultured with effector cells at an effector: target ratio of 3:1 for 72 h, the numbers of alive cells were counted. 

### 4.6. Cytokine Assay

To measure the production level of IL-7, non-transduced T cells, NKG2D-CAR or NKG2DIL7-CAR T cells (3 × 10^5^ cells per well) were cultured in 24-well plates with and without NKG2DLs^+^ PC-3 cells in an effector to target ratio of 2:1 without the addition of exogenous cytokines. After 24 h of co-culture, supernatants were harvested to measure IL-7 with enzyme-linked immunosorbent assay kit (Invitrogen, Carlsbad, CA, USA).

To detect the in vivo level of cytokine production, the mouse blood was collected and clotted at 4 °C, the sera were used to determine INF-γ, TNF-α, IL-6, IL-8, IL-1β and IL-12p70 with Human Inflammatory Cytokine Kit (BD Biosciences) according to the manufacturer’s instruction.

### 4.7. T-Cell Proliferation, Survival and Apoptosis Assay

To assess T-cell growth, survival and apoptosis upon antigen exposure, non-transduced T cells, NKG2D-CAR or NKG2DIL7-CAR T cells were labeled with CFSE (2 µM) and co-cultured with the target cells PC-3 in a ratio E:T (3:1) without the addition of exogenous cytokines. After 7 days of co-culture, CFSE dilution was measured for T cell division. Annexin-V/7-amino-actinomycin (7-ADD) staining was used to determine the apoptotic rate and the expression of Glut-1, Bcl-2 and exhaustion markers such as PD-1 and Tim-3 was detected by FACS analysis. 

### 4.8. Quantitative Real-Time PCR

Effector and target cells were co-cultured in E:T (2:1) for 24 h and the total RNAs were extracted from cultured cells using Trizol reagent (Takara) and reverse-transcribed using Reverse Transcription Kit (Prime Script First Strand cDNA Synthesis kit R047A, Takara). Reverse-transcribed single-stranded DNA was subjected to quantitative real-time PCR (q-PCR) using SYBR green master mix and amplified on Light Cycle (Agilent). Each experiment was performed in a duplicate manner and relative expression was calculated using the 2^−ΔΔCt^ change-in-cycling-threshold method with GAPDH as a reference. Primers were designed using primer 5 as follows: Glut1, sense, 5′-ATTGGCTCCGGTATCGTCAAC-3′, antisense, 5′-GCTCAGATAGGACATCCAGGGTA-3′; GAPDH, sense, 5′-AGGTCGGTGTGAACGGATTTG-3′, antisense, 5′-TGTAGACCATGTAGTTGAGGTCA-3′.

### 4.9. Xenograft Model

Female 6- to 8-week-old NOD/SCID/γ-chain^−/−^ (NSG) mice, purchased from Beijing Biocytogen Co., Ltd., were raised, treated and maintained in a non-specific pathogenic environment under the approval of the Animal ethics Committee of East China Normal University. To establish a prostate cancer model, mice were inoculated subcutaneously with 2 × 10^6^ PC-3 cells on its right flank and defined it as day 0. Mice were observed regularly, and their tumor dimensions were measured with calipers. When the tumor burden reached approximately 150–200 mm^3^, animals were randomly divided into three study groups (*n* = 6) and injected intravenously (i.v.) with 200 µL of T cells, NKG2D-CAR and NKG2DIL7-CAR T cells (1 × 10^7^ cells/mouse). No exogenous cytokines were injected in the mice. The magnitudes of tumors were measured by caliper and the volume of tumors was calculated using formula V = π/6 × (length × width^2^), where length is the largest longitudinal diameter and width is the largest transverse diameter. Mouse survival was observed and mice were sacrificed when the tumor burden reached a size of 1500–2000 mm^3^. 

### 4.10. Histopathological Analysis

To assess histopathological changes, tumor tissues were fixed with 4% paraformaldehyde and embedded in paraffin wax. Tissues were sliced into 4 μm-thick tumor sections and then stained with hematoxylin/eosin (H&E) for visualization of the tissue structure. For the IHC staining assay, the tissue sections were deparaffinated and incubated with 5% bovine serum albumin for 60 min. Then the tissue sections were incubated with anti-CD3 antibody (1:100, Servicebio, GB130144-M), anti-CD4 antibody (1:100, Servicebio, GB13064) or anti-CD8 antibody (1:100, Servicebio, GB13068) for 2 h, respectively. After incubation, 3% H_2_O_2_ was used to eliminate the activity of endogenous peroxidase and Goat anti-rabbit lgG was used as a secondary antibody. 3,3′-diaminobenzidinetetrahydrochloride (DAB-4HCl) was used to visualize the CD3, CD4 and CD8 expression. Images were acquired using a Nikon Eclipse 80i microscope (Nikon, Badhoevedorp, The Netherlands). Tissue evaluation was performed by two independent examiners and semi quantitated by image J Software.

### 4.11. Statistical Analysis

The data were reported as mean ± SD. Statistical analysis was performed using unpaired Student’s *t*-test or ANOVA. Survival was plotted using a Kaplan−Meier survival curve and statistical significance was determined by the Log-rank (Mantel−Cox) test. Prism software version 6.0 (GraphPad) was used for statistical calculation and *p*-value < 0.05 was accepted as indicating a significant difference.

### 4.12. Ethics Approval and Consent to Participate

All fresh blood was collected under a protocol approved by the Ethics Committee of East China Normal University, following written informed consent. All animal studies were approved by the Institutional Animal Care and Use Committee of East China Normal University (Approval No. m20170224).

## 5. Conclusions

In conclusion, our study emphasized the potential of NKG2D-based CAR T cells as a promising therapeutic option for prostate cancer. The incorporation of stimulation cytokine IL-7 could further improve the expansion and antitumor effects of NKG2D-CAR T cells and increase its potential clinical applicability. In addition, the strategy of co-expression of IL-7 can also be used to modify other targeted CAR T cells and to the treatment of other solid tumors.

## Supplementary Materials

The following are available online at https://www.mdpi.com/2072-6694/12/7/1969/s1, Figure S1: Expression level of NKG2D ligands. Q-PCR (a) and flow cytometry analysis (b) of the expression of NKG2D ligands on prostate cancer cell lines of PC-3, DU 145 and C4-2. The same cells stained with isotype antibody were used for gating (black). Data shown are representatives of experiments with similar results, Figure S2: Expression level of NKG2D CAR. Human T cells were transduced with lentiviruses expressing NKG2D-CAR or NKG2DIL7-CAR and the representative flow cytometry plot was indicated. Gating was based on the same cells stained with isotype-matched antibody. Data shown are representatives of experiments with similar results.

## Abbreviations

7-AAD	7-Aminoactinomycin D;
CAR	chimeric antigen receptor;
E:T ratio	effector-to-target ratio;
Glut1	glucose transporter 1;
GzmB	granzyme B;
IFN-γ	interferon gamma;
mCRPC	metastatic castration-resistant prostate cancer;
NKG2D	natural killer group 2 member D;
NKG2DLs	NKG2D ligands;
NT	non-transduced T cells;
PBMC	peripheral blood mononuclear cells;
q-PCR	quantitative real-time PCR;
TNF-α	tumor necrosis factor alpha.
