# Supplementary material for: Co-Expression of IL-7 Improves NKG2D-Based CAR T Cell Therapy on Prostate Cancer by Enhancing the Expansion and Inhibiting the Apoptosis and Exhaustion
Source: Cancers (Basel). 2020 Jul 20;12(7):1969. doi: 10.3390/cancers12071969 (PMC7409228; doi:10.3390/cancers12071969)
Supplement: Supplementary file 1 [file cancers-12-01969-s001.pdf]

cancers

Article

Supplementary Materials:

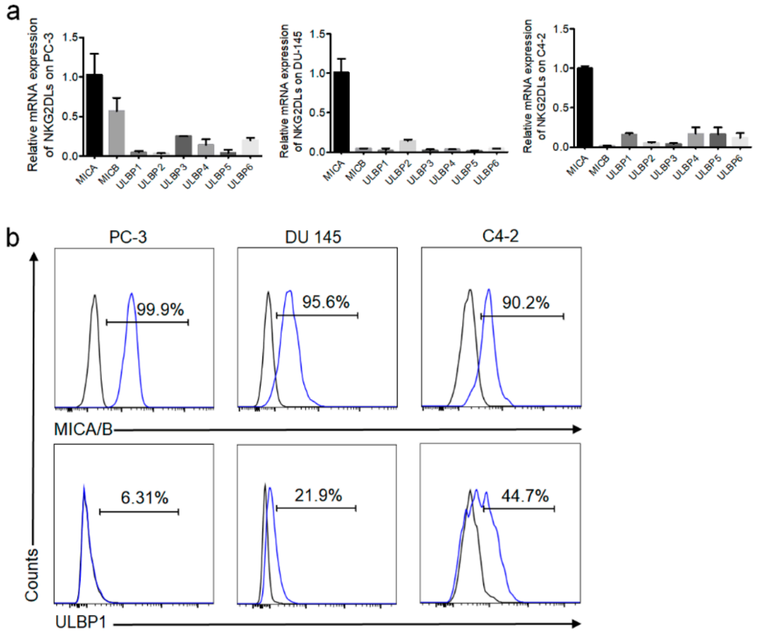

Figure S1. Expression level of NKG2D ligands. Q-PCR (**a**) and flow cytometry analysis (**b**) of the expression of NKG2D ligands on prostate cancer cell lines of PC-3, DU 145 and C4-2. The same cells stained with isotype antibody were used for gating (black). Data shown are representatives of experiments with similar results.

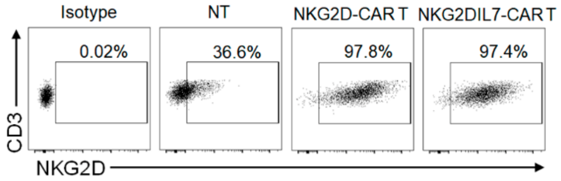

Figure S2. Expression level of NKG2D CAR. Human T cells were transduced with lentiviruses expressing NKG2D-CAR or NKG2DIL7-CAR and the representative flow cytometry plot was indicated. Gating was based on the same cells stained with isotype-matched antibody. Data shown are representatives of experiments with similar results.

© 2020 by the authors. Licensee MDPI, Basel, Switzerland. This article is an open access article distributed under the terms and conditions of the Creative Commons Attribution (CC BY) license (http://creativecommons.org/licenses/by/4.0/).
